# Carbapenem-resistant *Enterobacteriaceae* in sink drains of 40 healthcare facilities in Sindh, Pakistan: A cross-sectional study

**DOI:** 10.1371/journal.pone.0263297

**Published:** 2022-02-03

**Authors:** Paschal A. Apanga, Jamil Ahmed, Windy Tanner, Katherine Starcevich, James A. VanDerslice, Ubed Rehman, Najeebullah Channa, Scott Benson, Joshua V. Garn

**Affiliations:** 1 Department of Epidemiology, Biostatistics, and Environmental Health, School of Public Health, University of Nevada, Reno, Nevada, United States of America; 2 US- Pakistan Center for Advanced Studies in Water, Mehran University of Engineering and Technology, Jamshoro, Pakistan; 3 Department of Family and Preventive Medicine, University of Utah, Salt Lake City, Utah, United States of America; 4 Yale School of Public Health, New Haven, Connecticut, United States of America; Government College University Faisalabad, PAKISTAN

## Abstract

In Pakistan, antimicrobial resistance (AMR) is expected to greatly increase the already high mortality and morbidity rates attributed to infections, making AMR surveillance and prevention a priority in the country. The aims of the project were to characterize the prevalence of carbapenem-resistant *Enterobacteriaceae* (CRE) in healthcare facility sink drains in Pakistan and to characterize how physical characteristics of sinks and healthcare facility rooms were associated with CRE in those sinks. The study took place in 40 healthcare facilities in Jamshoro Pakistan. Swabs were collected from sink drains in each facility that had a sink, and structured observations of sinks and facilities were performed at each facility. Swabs were plated on CHROMagar KPC to screen for carbapenem-resistant *Enterobacteriaceae*, which were then isolated on Mueller-Hinton agar plates. Antibiotic susceptibility was determined using the disk diffusion method to assess resistance to carbapenems, cephalosporins, and fluoroquinolones. Thirty-seven of the healthcare facilities had at least one sink, and thirty-nine total sinks were present and sampled from those healthcare facilities. Sinks in these facilities varied in quality; at the time of sampling 68% had water available, 51% had soap/alcohol cleanser at the sink, 28% appeared clean, and 64% drained completely. Twenty-five (64%) of the sink samples grew *Enterobacteriaceae* on CHROMagar KPC, sixteen (41%) of which were clinically non-susceptible to ertapenem. Seven of the 39 sampled sinks (18%) produced *Enterobacteriaceae* that were resistant to all three antibiotic classes tested. Several facilities and sink characteristics were associated with CRE. Sinks and drains can serve as undetected reservoirs for carbapenem-resistant *Enterobacteriaceae*. Control and remediation of such environments will require both systemic strategies and physical improvements to clinical environments.

## Introduction

Over the past decade, there has been a rapid increase in antimicrobial resistance (AMR), especially multi-drug resistance, and this has led to decreased effectiveness of available antimicrobials leading to an increase in AMR-associated infection worldwide, and particularly in south Asian countries such as Pakistan [1–3]. Due to this rapid increase, AMR has become a significant cause of morbidity and mortality globally [3, 4]. Currently, over 700,000 deaths globally are attributed to antibiotic-resistant infections each year [5]. It is estimated that by 2050, a cumulative 100 trillion United States Dollar (USD) of economic costs and 10 million lives a year are at risk due to the rise of drug-resistant infections with most of the impact falling on low-and middle-income countries [5]. In Pakistan, AMR is expected to greatly increase the already high mortality and morbidity rates attributed to infections [6], making AMR surveillance and prevention a priority in the country [1, 2].

Several factors have contributed to the emergence of AMR in Pakistan, including simultaneous use of multiple drugs to treat conditions, misleading advertising, irrational prescribing, availability of over-the-counter drugs without prescription, and widespread use of antibiotics in poultry, other animals, and agriculture [2, 6, 7]. Klein et al. found that between the years 2000 and 2015, total human antibiotic consumption in Pakistan increased by 65%, from 800 million defined daily doses (DDD) to 1.3 billion DDD [6]. Previous research has demonstrated a direct relationship between the consumption of antibiotics and the development and spread of resistant strains [8], making the high consumption of antibiotics in Pakistan of particular concern.

A lack of surveillance has also contributed to AMR in Pakistan [1]. To address this gap, Pakistan began the implementation of a national AMR surveillance system in 2015 [1]. Despite this system, an accurate estimate of the prevalence of nosocomial (hospital-acquired) infections in Pakistan is unknown, and the prevalence of resistant infections is expected to be substantial in public sector hospitals [6]. The risk of nosocomial resistant bacterial infection in Pakistan may be as high as 60% [6].

In healthcare settings, carbapenem-resistant microorganisms are of particular concern [9]. Carbapenems are broad spectrum microbial agents highly effective against Gram-positive, Gram-negative, and anaerobic bacteria [9]. Due to their potency, carbapenems are used as a “last resort antimicrobial” [9]. Carbapenem-resistant *Enterobacteriaceae* (CRE) are of particular concern in hospital settings due to their high resistance to carbapenems and association with high rates of morbidity and mortality [10]. Resistance to multiple classes of antibiotics increases the risk of morbidity and mortality, as it limits or eliminates effective antimicrobial treatment options [3]. In Pakistan, carbapenem-resistant bacteria have been found in multiple hospitals, including infections caused by *Klebsiella pneumoniae* and *Acinetobacter baumannii* [11, 12]. The emergence and increase in carbapenem-resistant nosocomial and community infections has necessitated increased monitoring of carbapenem-resistant microorganisms and associated infections [12].

Frequent hand washing is essential for infection prevention [5], however outbreaks of multidrug-resistant bacterial infections, including CRE, have been linked to contaminated hand hygiene sinks in healthcare facilities [13–15]. In previous studies, researchers found that the physical design of the sink, physical components of sinks including drain configuration [14–16] and sink depth [13], contributed to the formation of biofilm in the tailpiece and trap as well as the degree of splashing and subsequent spread of bacteria in droplets. Other practices, such as disposal of bodily fluids and medications down sink drains and the frequency of cleaning have also been associated with antibiotic-resistant bacteria (ARB) growth [15].

The primary aim of the study was to characterize the prevalence of carbapenem-resistant *Enterobacteriaceae* in sink drains in health facilities in the Jamshoro district of Pakistan. A secondary aim was to assess the resistance of carbapenem-resistant *Enterobacteriaceae* to other classes of antibiotics, including cephalosporins, beta-lactams, and fluoroquinolones. The final aim was to determine how water, sanitation and hygiene (WASH) practices, and how physical characteristics of sinks and healthcare facility rooms are associated with the presence of CRE in those sinks.

## Methods

### Study design and setting

A cross-sectional survey assessing WASH indicators and testing sink drains for carbapenem-resistant *Enterobacteriaceae* was conducted in healthcare facilities in Jamshoro district in the Sindh province of Pakistan. The data were collected between March 3^rd^ and May 9th, 2019. Jamshoro district has a population of nearly one million people, served by 69 healthcare facilities [17, 18].

#### Healthcare facility sampling

Eligible healthcare facilities included primary, secondary, and tertiary facilities as defined by the Sindh Health Department [17]. Stratified random sampling was used to select primary and secondary healthcare facilities equally. However, because there was only one (1) tertiary healthcare facility in Jamshoro district, it was also included in the study. In total, forty (40) healthcare facilities were selected.

#### Data collection

An adult employee ≥18 years of age with the knowledge of WASH indicators in the facility was interviewed from each facility. Questions on sinks and other WASH indicators were adapted from World Health Organization/ United Nations Children’s Fund (WHO/UNICEF) core questions and indicators for monitoring WASH in health care facilities [19]. Observations of the physical characteristics of the facilities and environmental samples from sinks were also collected. Surveys were conducted using Open Data Kit [20]. In addition, photos were taken of sinks sampled. Prior to sampling, pilot testing was conducted in five of the healthcare facilities that were not selected to be in our study, to pretest the sampling procedures, the survey, and the laboratory testing. Those pilot data were not included in these analyses.

#### Sink sampling

Thirty-seven (37) of the 40 facilities in the study had at least one sink. Sinks were sampled from all facilities that had at least one sink. Flocked Eswabs (COPAN Diagnostics, Murrieta, CA) were used to collect samples from sink drains. The swab was inserted (up to the red break line on the swab) into the drain and then rotated against the side of the sink pipe wall several times until half of the circumference of the drainpipe had been sampled. The collected samples were preserved in tubes with Amies medium and transported on ice to the laboratory for culture and isolation of carbapenem-resistant *Enterobacteriaceae*, followed by antimicrobial susceptibility testing.

#### Laboratory methods

Swabs were processed the same day the sample was collected. Swabs were vortexed and sterilized forceps were used to remove the swab from the tube. Ten microliters was pipetted from the one milliliter of vortexed transport media onto CHROMagar KPC plates (DRG International, Springfield, New Jersey), which contained a proprietary supplement that selects for carbapenem-resistant bacteria. The liquid was spread around the surface of the agar plate, and the plates were incubated at 37°C overnight. The following day the plates were inspected for *E*. *coli* and coliform growth and if a rose-colored colony (representative of *E*. *coli*) was present, it was then selected and streaked to Mueller Hinton agar for isolation. If *E*. *coli* colonies were not present, a dark blue coliform colony, representative of other *Enterobacteriaceae* species, was instead selected and streaked to Mueller Hinton agar for isolation. We prioritized *E*. *coli* over total coliform bacteria was because *E*. *coli* is more likely to cause illness. If colonies were too closely mixed, colony clusters were selected and streaked to a new CHROMagar KPC plate to isolate colonies. Individual colonies were selected from 18 to 24-hour old Mueller Hinton plates to make a suspended solution equivalent to a 0.5 McFarland standard. Clinical antibiotic resistance to the selected antibiotics was determined by the antibiotic disk diffusion method. Briefly, the solution was plated on Mueller Hinton agar plates and ertapenem, ceftriaxone, and ciprofloxacin disks were placed on the plates After an 18–24 hour incubation, calipers were used to measure disk zones of inhibition which were compared to CLSI antimicrobial breakpoints to determine the susceptibility of isolates to each antibiotic [21].

#### Outcomes

The primary outcome of interest was the presence/absence of carbapenem-resistant *Enterobacteriaceae* in healthcare facility sink drains. Sink swabs were plated on CHROMagar KPC to screen for carbapenem-resistant and *Enterobacteriaceae* isolates were classified as carbapenem-non-susceptible if they exhibited an ertapenem zone of inhibition of less than 22 mm. The secondary outcome of interest was the prevalence of carbapenem-resistant *Enterobacteriaceae* demonstrating additional resistance to other classes of antibiotics including cephalosporins (i.e., ceftriaxone) and fluoroquinolones (i.e., ciprofloxacin).

*WASH and health care indicators*. Enumerators used structured observations to collect data on various facility and sink characteristics, which were thought to plausibly be associated with the presence of ARB in or around sinks. Facility level variables that were collected included: the facility’s water source type (piped water, borehole, tube well, dug well, spring water, surface water, or water from a tanker truck); the facility’s care level (primary, secondary, tertiary); and whether the facility had separate functional hand hygiene stations aside from the sinks (yes/no). These hand hygiene stations are different from sinks and may include the use of a pan with soap and water or a dispenser with an alcohol-based hand rub. Physical characteristics of the sinks that were collected included: sink location (general ward, outpatient clinic, or dispensary); water availability at sink (yes/no); soap/alcohol cleanser available at sink (yes/no); drying implements available at sink (yes/no); who cleaned the sink (staff vs others); sink cleanliness (yes/no), whether the sink drained completely (yes/no), whether the tap handles stop the flow of water completely (yes/no); whether patients used the sink (yes/no); and the presence of a counter around sinks (yes/no). Finally, uses of the sinks included: disposing of cleaning products in the sink (yes/no), disposing of human waste in the sink (yes/no), using sink water to clean the room (yes/no), washing medical supplies in sink (yes/no), and patients using sink water for drinking (yes/no).

### Statistical analysis

All data were cleaned and analyzed using SAS software version 9.4. Descriptive statistics were used to characterize the healthcare WASH facilities and the characteristics of sinks in the healthcare facilities in the study. A bivariate analysis was conducted to determine if there were any differences between any of the characteristics of interest, and the prevalence of ARB growth in sinks. Differences between exposures and specific antimicrobial resistance were tested for significance using a mid-p exact test; p-values of less than 0.05 were considered statistically significant.

### Ethical consideration

Ethical approval was obtained from the Mehran University of Engineering and Technology, Institutional Review Board, in Jamshoro, Pakistan. The University of Nevada, Reno received de-identified data from US- Pakistan Center for Advanced Studies in Water, Mehran University of Engineering & Technology. Although this was an environmental study that did not collect data on humans, we did interview employees asking questions about WASH and other conditions at participating facilities, and verbal consent was required from these participating employees in order for the site to be eligible to be included in our study.

## Results

### Prevalence of resistant *Enterobacteriaceae*

Thirty-seven of the 40 facilities had at least one sink. Thirty-nine sinks were located in and sampled from the 37 sampled healthcare facilities. Of the 39 sink samples, 25 (64%) screened positive for carbapenem-resistance having at least one colony (presence) of *Enterobacteriaceae* growth on the CHROMagar KPC (Table 1). Ten of these samples produced bacteria categorized as *Escherichia coli*, as determined by colony appearance and color on chromogenic media; 15 of the remaining samples produced bacteria that were categorized as total coliform bacteria *(i*.*e*., *Enterobacteriaceae* that were not *E*. *coli*). When tested for resistance to ertapenem by the disk diffusion method, 16 samples were considered clinically non-susceptible to ertapenem (8 *E*. *coli* and 8 total coliforms). *Enterobacteriaceae* from 24 of the 25 samples were non-susceptible to at least one other class of antibiotics (S1 Table). Isolates from 7 of the 25 positive samples (28%) exhibited resistance to all tested antibiotics, representing 18% of the 39 sinks sampled in this study.

**Table 1:** Antibiotic resistance patterns of 39 sinks from 37 healthcare facilities in Sindh Pakistan.

	N	%
*Enterobacteriaceae* that screened positive for carbapenem resistance on CHROMagar KPC	25	*64%*
*E-coli* that screened positive on CHROMagar KPC	10	
Non-susceptible to ertapenem	8	*21%*
Non-susceptible to ceftriaxone	7	*18%*
Non-susceptible to ciprofloxacin	1	*3%*
Total coliforms that screened positive on CHROMagar KPC	15	
Non-susceptible to ertapenem	8	*21%*
Non-susceptible to ceftriaxone	13	*33%*
Non-susceptible to ciprofloxacin	13	*33%*

### Predictors of carbapenem-resistant *Enterobacteriaceae*

Most (87%) of the facilities in our study were primary health care facilities (Table 2). Water sources in these facilities were most frequently piped from off site (64%), with 26% of facilities having their own borehole and 10% of facilities supplied by a tanker truck. Sinks across all facilities varied in quality, with 68% having water available at the time of the visit, 51% having soap/alcohol cleanser at the sink, 28% of sinks appearing clean, and 64% of sinks draining completely (Table 2).

**Table 2:** Association between facility and sink characteristics and screening positive for carbapenem-resistant *Enterobacteriaceae* growth on CHROMagar KPC in 39 sinks from 37 healthcare facilities in Sindh Pakistan.

	All facilities		Sinks with ertapenem-resistant growth		p-value
	N = 39	% of total	N positive	% positive	
**Facility Characteristics**					
Care Level					0.754
Primary	34	87%	22	65%	
Secondary	4	10%	2	50%	
Tertiary	1	3%	1	100%	
Facility water source					**0.015**
Piped from off-site source	25	64%	20	80%	
Borehole	10	26%	3	30%	
Tanker truck	4	10%	2	50%	
Facility has hand hygiene stations besides main sink	3	8%	0	0%	**0.020**
No separate hand hygiene stations	36	92%	25	69%	
**Sink Characteristics**					
Sink location*					0.186
General ward	7	19%	6	86%	
Dispensary	7	19%	6	86%	
Outpatient clinic	22	59%	11	50%	
Other	1	3%	1	100%	
Water available at sink*	25	68%	18	72%	**0.048**
Water unavailable	12	32%	5	42%	
Soap/alcohol cleanser available at sink	20	51%	11	55%	0.256
Soap/alcohol cleanser unavailable	19	49%	14	74%	
Water available and soap/alcohol available at sink*	9	25%	6	67%	0.847
Water and soap/alcohol unavailable	27	75%	17	63%	
Drying implements available at sink	2	5%	0	0%	0.053
Drying implements unavailable at sink	37	95%	25	68%	
Sink is reported to be cleaned by staff	35	90%	23	66%	0.443
Not cleaned	4	10%	2	50%	
Sink appears clean	11	28%	4	36%	**0.020**
Sink does not appear clean	28	72%	21	75%	
Sink drains completely	25	64%	15	60%	0.620
Sink does not drain	14	36%	10	71%	
Tap handles stop flow of water completely	26	67%	17	65%	0.867
Water drips	13	33%	8	62%	
Cleaning products disposed of in sink	24	62%	16	67%	0.618
Cleaning products not disposed of in sink	15	38%	9	60%	
Human waste disposed of in sink	2	5%	2	100%	0.325
Human waste not disposed of in sink	37	95%	23	62%	
Counter around sink	6	15%	6	100%	**0.043**
No counter around sink	33	85%	19	58%	
Sink water used to clean room and sinks	14	36%	11	79%	0.134
Sink water not used to clean	25	64%	14	56%	
Medical supplies washed in sink	3	8%	2	67%	0.770
Medical supplies not washed in sink	36	92%	23	64%	
Sink water used as patient drinking water	1	3%	1	100%	0.680
Sink water not used as drinking water	38	97%	24	63%	

**Bold** = statistically significant p<0.05. Exact test mid-p value was used for all analyses.

*This variable contains missingness.

**Fig 1:**
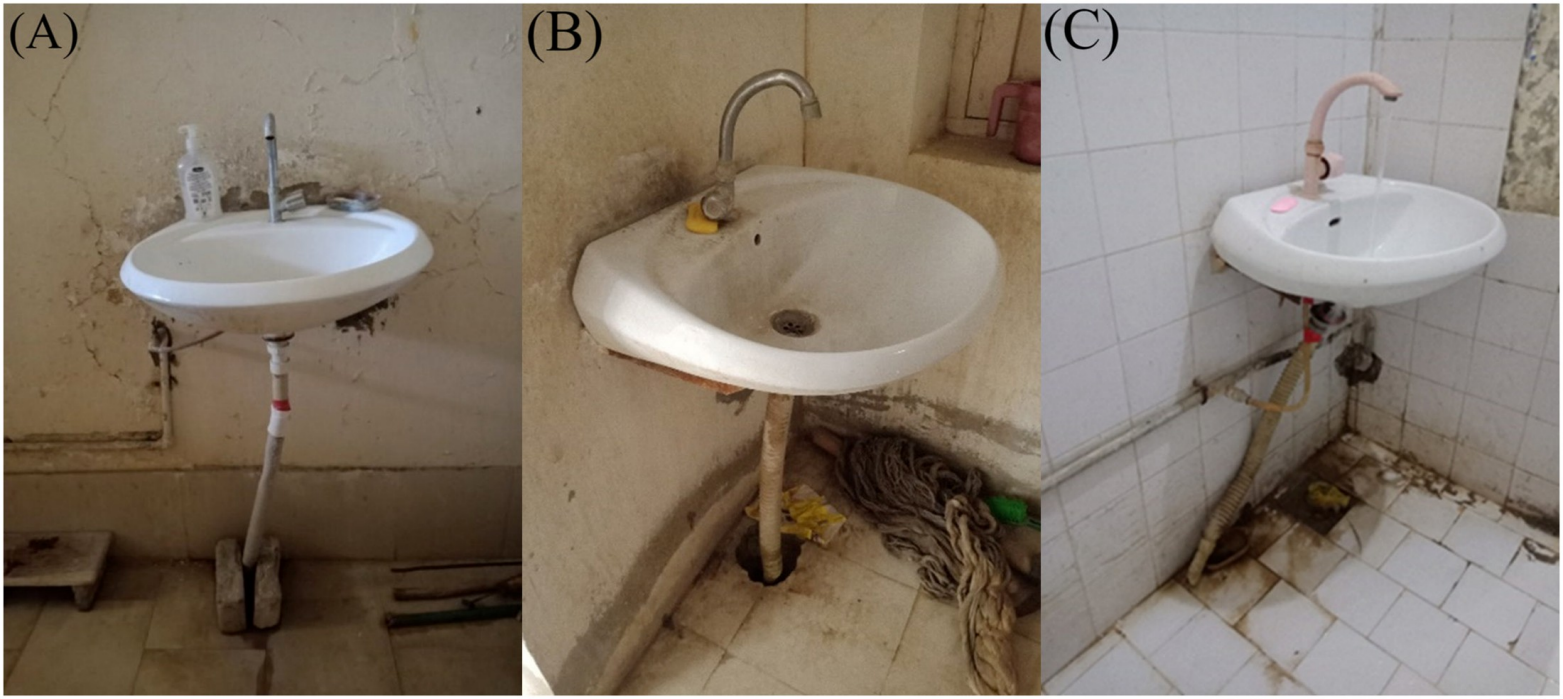
A) Sink with straight drainpipe with leak at floor. B) Dirty sink and room. C) Sink with a pipe disconnected both at drain and floor, with water spilling directly onto the floor.

Several characteristics specific to the facilities and sinks were associated with ertapenem resistance (Table 2) and with additional resistance to specific antibiotics (S2–S4 Tables). *Enterobacteriaceae* screening positive for carbapenem resistance were more likely to be found in facilities that had had water piped from the outside than in facilities that had a borehole (*p* = 0.015; Table 2). Similarly, piped water was associated with increased non-susceptibility to ceftriaxone (S2 Table). *Enterobacteriaceae* screening positive for carbapenem resistance were more common in sinks that had water available at the sink the day of the visit (*p* = 0.048; Table 2), and in dirty sinks (*p* = 0.020; Table 2). In addition, sink cleanliness was associated with increased non-susceptibility to ciprofloxacin (S3 Table). Facility characteristics, including having a countertop surrounding the sink (*p* = 0.043) and the facility having separate functional hand hygiene stations at points of care (*p* = 0.020) were both associated with screening positive for carbapenem resistance (Table 2). The sinks were not observed to have been plumbed with p-traps (none of our photographs or observations found a p-trap). In some sinks, the drainpipe discharged directly onto the floor and in others the sink was not fitted with a drainpipe causing discharge directly onto the floor (Fig 1). Fig 1(A) shows a straight drainpipe and a floor that shows evidence of leaking from that pipe onto the floor. Fig 1(B) shows a dirty sink and room, with a pipe that drains into the ground. Fig 1(C) shows a pipe that is completely disconnected from both the sink base and also the floor, and shows running water spilling from the sink base onto the floor.

## Discussion

In our study of 40 healthcare facilities in Sindh, Pakistan, 25 (64%) of the sampled sinks displayed *Enterobacteriaceae* growth on CHROMagar KPC plates selective for carbapenem-resistant bacteria. Sixteen of these were non-susceptible to ertapenem, a “last-resort” antimicrobial. Seven of the 25 samples that were positive for ertapenem-resistant *Enterobacteriaceae* growth also demonstrated additional resistance to the other classes of antibiotics. AMR can be either inherent in the organism or acquired though gene transfer or acquisition from the surrounding environment or other microorganisms [22]. Our findings of CRE in sinks may be a reflection of organisms that patients and/or caregivers are harboring, and also may represent a potential source of ARB for the hospital environment, patients, caregivers and guests, particularly due to potential contamination of the area surrounding the sink due the lack of enclosed drains. While understanding and anticipating inherent resistance by certain species of microorganisms is necessary for proper antibiotic selection, the continued pressure and evolution of multidrug-resistant organisms make initial antibiotic selection complicated and endangers the care of patients, threatens the utility of antibiotics, and increases the cost of medical care.

The high prevalence of bacteria resistant to antibiotics of “last-resort” in our study is of high concern, and there are several factors that suggest that our results might even be underestimated. First, we had much lower prevalence of detection of resistant organisms among sinks that did not have water available at the time of sampling (42.0% vs. 72.0%). Since the presence of water facilitates bacterial growth [23], we likely would have observed higher prevalence of ARB had all of the sinks had water available at the time of sampling. Second, the sinks we assessed usually had a straight drainpipe (Fig 1) and sometimes no drainpipe at all, rather than having a standardized p-trap, and p-traps create an environment that is more conducive for trapping and colonizing bacteria [16, 24, 25].

The spread of infectious organisms from contaminated sinks in healthcare facilities endangers patients already experiencing comorbid conditions. Eliminating contamination spreading from the sinks externally could potentially reduce the spread of these organisms and prevent further growth, contamination and infection [1]. Other studies have shown potential spread of ARB from sinks through splashing onto countertops [13, 15]. In our study, countertops were rare, but CRE growth was present in all 6 sinks with countertops. Some sinks in this study lacked proper drainage pipes or connections and were observed to drain directly onto the floor, leading to high potential for localized contamination of floors and surrounding surfaces with ARB. The spread of contamination throughout the room may be more feasible than trying to eliminate the contamination within the sink. Prior studies found that daily and twice daily cleaning of sinks was ineffective in eliminating and preventing biofilm formation [14, 15]. Intervention strategies that alter physical characteristics of sinks to ensure timely draining and minimize backsplash have demonstrated more effective in the prevention of AMR spread [13–15]. and should be considered in this context. Improving drain designs would also likely be beneficial in this particular setting.

Facility water source was significantly associated with resistance to ertapenem and ceftriaxone (i.e., a broad-spectrum antibiotic). The source of the ertapenem-resistance is not clear from our study. According to governmental reports in Pakistan, 81% of municipal water sources tested in Sindh province were unsafe for human consumption due to bacterial contamination [26]. Contaminated water supplies have been found to be a source of antibiotic-resistant bacteria [27], and waterborne nosocomial infection [28], although it is not clear if this is a source the CRE contamination in our study.

The study has several limitations. Our study had a relatively small sample size of healthcare facilities, and not all of those health care facilities had sinks. The results should not be generalized to healthcare settings in other countries, given that some of the facilities and sink characteristics that we observed are likely distinctive to this setting in Pakistan. In addition, the culture methods we used in our study relied on chromogenic colony selection and did not allow for the identification of bacterial strains. Finally, due to our cross-sectional design, the associations that we observed between sink and facility characteristics and ARB may not represent causal relationships.

## Conclusions

We found a high prevalence of ertapenem-resistant bacteria in sink drains in health facilities in the Jamshoro district of Pakistan. This study also demonstrates that sink characteristics, design and functionality might play a vital role in sink drains acting as a reservoir for ARB in health care facilities in low and middle-income countries. Regardless of whether ARB are introduced into the sink environment through the water source, patient care, hand cleaning or use of the sink for waste disposal the potential for spread of ARB from sinks (and particularly sink drainage in this setting) to the surrounding facility appears to be high. In addition to systems controls such as antibiotic stewardship programs and appropriate use and disposal of antibiotics, physical facilities, such as sinks and countertops, and drainage systems must be designed and maintained to reduce or eliminate potential environments for ARB in health care facilities. Our study also highlights the need to improve infrastructure to achieve handwashing in healthcare facilities in the Jamshoro district of Pakistan.

## Supporting information

S1 Table Antibiotic resistance patterns of 25 samples screening positive for carbapenem-resistant *Enterobacteriaceae* growth among 39 sinks from 37 healthcare facilities in Sindh Pakistan. (DOCX)

S2 Table Association between facility and sink characteristics and ceftriaxone resistance or intermediate resistance in 39 sinks from 37 healthcare facilities in Sindh Pakistan. (DOCX)

S3 Table Association between facility and sink characteristics and ciprofloxacin resistance or intermediate resistance in 39 sinks from 37 healthcare facilities in Sindh Pakistan. (DOCX)

S4 Table Association between facility and sink characteristics and ertapenem resistance or intermediate resistance in 39 sinks from 37 healthcare facilities in Sindh Pakistan. (DOCX)
