# Supplementary material for: Carbapenem-resistant Enterobacteriaceae in sink drains of 40 healthcare facilities in Sindh, Pakistan: A cross-sectional study
Source: PLoS One. 2022 Feb 3;17(2):e0263297. doi: 10.1371/journal.pone.0263297 (PMC8812900; doi:10.1371/journal.pone.0263297)
Supplement: S1 Table — (DOCX) [file pone.0263297.s001.docx]

**S1 Table.** Antibiotic resistance patterns of 25 samples screening positive for carbapenem-resistant Enterobacteriaceae growth among 39 sinks from 37 healthcare facilities in Sindh Pakistan.

| Pos. sample number | Isolate type | Ertapenem  non-susceptible | Ceftriaxone non-susceptible | Ciprofloxacin non-susceptible |
| --- | --- | --- | --- | --- |
| 1 | FC | Y | Y | Y |
| 2 | E-coli | Y | Y |  |
| 3 | FC | Y | Y | Y |
| 4 | E-coli | Y | Y |  |
| 5 | E-coli | Y |  |  |
| 6 | FC | Y | Y | Y |
| 7 | FC | Y | Y | Y |
| 8 | E-coli |  |  |  |
| 9 | E-coli | Y | Y |  |
| 10 | FC |  | Y | Y |
| 11 | E-coli | Y | Y |  |
| 12 | FC |  |  | Y |
| 13 | FC |  |  | Y |
| 14 | E-coli | Y | Y |  |
| 15 | E-coli | Y | Y |  |
| 16 | FC | Y | Y |  |
| 17 | FC |  | Y | Y |
| 18 | FC |  | Y | Y |
| 19 | FC |  | Y |  |
| 20 | E-coli | Y |  |  |
| 21 | FC | Y | Y | Y |
| 22 | FC | Y | Y | Y |
| 23 | FC | Y | Y | Y |
| 24 | FC |  | Y | Y |
| 25 | E-coli |  | Y | Y |
