# Supplementary material for: Carbapenem-resistant Enterobacteriaceae in sink drains of 40 healthcare facilities in Sindh, Pakistan: A cross-sectional study
Source: PLoS One. 2022 Feb 3;17(2):e0263297. doi: 10.1371/journal.pone.0263297 (PMC8812900; doi:10.1371/journal.pone.0263297)
Supplement: S2 Table — (DOCX) [file pone.0263297.s002.docx]

**S2 Table.** Association between facility and sink characteristics and ceftriaxone resistance or intermediate resistance in 39 sinks from 37 healthcare facilities in Sindh Pakistan.

|  | Total | |  | Ceftriaxone growth | |  |  |
| --- | --- | --- | --- | --- | --- | --- | --- |
|  | N=39 | % of total |  | N positive  =20 | % positive |  | p-value |
| **Facility Characteristics** |  |  |  |  |  |  |  |
| Care Level |  |  |  |  |  |  | 0.470 |
| Primary | 34 | 87% |  | 18 | 53% |  |  |
| Secondary | 4 | 10% |  | 1 | 25% |  |  |
| Tertiary | 1 | 3% |  | 1 | 100% |  |  |
| Facility water source |  |  |  |  |  |  | **0.044** |
| Piped from off-site source | 25 | 64% |  | 16 | 64% |  |  |
| Borehole | 10 | 26% |  | 2 | 20% |  |  |
| Tanker truck | 4 | 10% |  | 2 | 50% |  |  |
| Facility has hand hygiene stations besides main sink | 3 | 8% |  | 0 | 0% |  | 0.053 |
| No separate hand hygiene stations | 36 | 92% |  | 20 | 56% |  |  |
| **Sink Characteristics** |  |  |  |  |  |  |  |
| Sink location* |  |  |  |  |  |  | 0.420 |
| General ward | 7 | 18% |  | 4 | 57% |  |  |
| Dispensary | 7 | 18% |  | 5 | 71% |  |  |
| Outpatient clinic | 22 | 59% |  | 9 | 41% |  |  |
| Other | 1 | 5% |  | 1 | 100% |  |  |
| Water available at sink* | 24 | 68% |  | 14 | 58% |  | 0.182 |
| Water unavailable | 12 | 32% |  | 4 | 33% |  |  |
| Soap/alcohol cleanser available at sink | 20 | 51% |  | 9 | 45% |  | 0.435 |
| Soap/alcohol cleanser unavailable | 19 | 49% |  | 11 | 58% |  |  |
| Water available and soap/alcohol available at sink* | 9 | 27% |  | 4 | 44% |  | 0.721 |
| Water and soap/alcohol unavailable | 27 | 73% |  | 14 | 52% |  |  |
| Drying implements available at sink | 2 | 5% |  | 0 | 0% |  | 0.071 |
| Drying implements unavailable at sink | 37 | 95% |  | 20 | 54% |  |  |
| Sink is reported to be cleaned by staff | 35 | 90% |  | 19 | 54% |  | 0.224 |
| Not cleaned | 4 | 10% |  | 1 | 25% |  |  |
| Sink appears clean | 11 | 28% |  | 4 | 36% |  | 0.228 |
| Sink does not appear clean | 28 | 72% |  | 16 | 57% |  |  |
| Sink drains completely | 25 | 64% |  | 12 | 48% |  | 0.621 |
| Sink does not drain | 14 | 36% |  | 8 | 57% |  |  |
| Tap handles stop flow of water completely | 26 | 67% |  | 14 | 54% |  | 0.864 |
| Water drips | 13 | 33% |  | 6 | 46% |  |  |
| Cleaning products disposed of in sink | 24 | 62% |  | 12 | 50% |  | 0.874 |
| Cleaning products not disposed of in sink | 15 | 38% |  | 8 | 53% |  |  |
| Human waste disposed of in sink | 2 | 5% |  | 2 | 100% |  | 0.359 |
| Human waste not disposed of in sink | 37 | 95% |  | 18 | 49% |  |  |
| Counter around sink | 6 | 15% |  | 5 | 83% |  | 0.137 |
| No counter around sink | 33 | 85% |  | 15 | 45% |  |  |
| Sink water used to clean room and sinks | 14 | 36% |  | 8 | 57% |  | 0.628 |
| Sink water not used to clean | 25 | 64% |  | 12 | 48% |  |  |
| Medical supplies washed in sink | 3 | 8% |  | 2 | 67% |  | 0.803 |
| Medical supplies not washed in sink | 36 | 92% |  | 18 | 50% |  |  |
| Sink water used as patient drinking water | 1 | 3% |  | 1 | 100% |  | 0.744 |
| Sink water not used as drinking water | 38 | 97% |  | 19 | 50% |  |  |

**Bold**=statistically significant p<0.05. Exact test mid-p value was used for all analyses. *This variable contains missingness.
